# The efficacy of synovectomy for total knee arthroplasty: a meta-analysis

**DOI:** 10.1186/s13018-018-0752-y

**Published:** 2018-03-12

**Authors:** Zi-qin Zhao, Jin Xu, Rui-lin Wang, Li-na Xu

**Affiliations:** Department of Pathology, Tianjin Hospital, No. 406 Jiefang South Road, Hexi District, Tianjin, 300211 People’s Republic of China

**Keywords:** Synovectomy, Total knee arthroplasty, Meta-analysis

## Abstract

**Background:**

Many studies have proposed synovectomy during total knee arthroplasty (TKA) to reduce pain after TKA. The aim of this study was to assess the outcomes of synovectomy for treating of TKA through a meta-analysis.

**Methods:**

Relevant clinical studies on synovectomy and without synovectomy were retrieved through searching the databases PubMed, Embase, Web of Science, and the Cochrane Central Register of Controlled Trials up to January 2018. Studies that investigated the comparison of pain scores, total blood loss, range of motion, functional Knee Society Scores (KSSs), clinical KSSs, and operating time and provided sufficient data of interest were included in this meta-analysis. Stata 12.0 was used for meta-analysis.

**Results:**

Ten randomized controlled trials (RCTs) were finally included in this meta-analysis. Final results indicated that there was no significant difference between the pain scores, range of motion, functional Knee Society Scores (KSSs), and clinical KSSs (*P* > 0.05). However, synovectomy was associated with an increase of the total blood loss compared to patients without synovectomy (weighted mean difference (WMD) = 116.71, 95% confidence interval (CI) 78.63, 154.79, *P* = 0.000). Pooled results indicated that synovectomy was associated with an increase of the operating time (WMD = 15.44, 95% CI 2.67, 28.21, *P* = 0.018).

**Conclusions:**

Current evidence indicates that synovectomy has no effects on the final clinical outcomes for patients undergoing TKA. It will increase the total blood loss and the operating time during TKA.

## Background

Synovial proliferation is a common intraoperative finding in patients with osteoarthritis (OA) or rheumatoid arthritis (RA) of the knee [1]. Synovectomy when in total knee arthroplasty (TKA) for primary OA, excising the inflamed or proliferated synovial membrane, reduces pain and improves joint function and is beneficial for patients with RA and other inflammatory disease [2]. Although many inflammatory cytokines have been isolated from the knee and the blood in patients with moderate to severe primary osteoarthritis, [3] the benefit of synovectomy as a sole procedure in OA remains unclear.

Most studies have reported favorable results following synovectomy combined with other specific surgical procedures [4]. Synovectomy undertaken during total knee arthroplasty (TKA) depends on the surgeon’s preference [5]. Krackow et al. [6] recommended that as little synovium as possible should be removed. Synovitis has been reported as one of several contributing factors in unsatisfactory results after TKA [7]. This raises the question as to whether intraoperative synovectomy during TKR would be advantageous in decreasing postoperative inflammation of the knee.

A previous meta-analysis compared synovectomy to without synovectomy for clinical outcomes in patients who underwent TKA [8]. However, the disadvantages were as follows: (1) only three studies were included and thus will increase the type I error probability; (2) operating time was not compared; and (3) additionally, more evidence is emerging, and it is necessary to re-evaluate the efficacy of synovectomy for clinical outcomes after TKA. This meta-analysis aimed to evaluate whether synovectomy can decrease pain intensity and increase clinical outcomes after TKA.

## Methods

### Literature search

The electronic databases PubMed, Embase, and Cochrane Central Register of Controlled Trials were searched for all articles on synovectomy and without synovectomy for treating OA patients. The following were the search terms: (synovectomy OR) AND (TKA OR TKR OR total knee arthroplasty OR total knee replacement OR “Arthroplasty, Replacement, Knee”[Mesh]) where the search date was January 2018. In addition, a manual search of the bibliographies of the identified articles was performed to elucidate potentially relevant studies. The reliability of the study selection was determined by Cohen’s kappa test, and the acceptable threshold value was set at 0.61 [9, 10].

### Inclusion and exclusion criteria

Abstracts of all citations and retrieved studies were reviewed. Studies meeting the following criteria were included: (1) patients prepared for TKA, (2) randomized controlled trial (RCTs), (3) intervention was synovectomy and control was without synovectomy for TKA, and (4) outcomes including pain scores, total blood loss, range of motion, functional Knee Society Scores (KSSs), clinical KSSs, and operating time.

Studies were excluded if one of the following existed: providing undefined sample and control source, non-therapeutic clinical studies, animal experiments, non-original studies, and undefined grouping.

### Quality assessment

The methodological quality of all included trials was independently assessed by two reviewers using the Cochrane Handbook for Systematic Reviews of Interventions, version 5.1.0 (http://handbook.cochrane.org/). A total of seven items (random sequence generation, allocation concealment, blinding to the participant and personnel, blinding to the outcome assessment, incomplete outcome, selective reporting, and other bias) were measured. Each of the items was measured as “low risk of bias,” “unclear risk of bias,” and “high risk of bias.” The risk of bias summary and risk of bias graph were obtained using Review Manager 5.3.0 software (The Nordic Cochrane Centre, The Cochrane Collaboration, Copenhagen, Denmark).

### Data extraction

A specific extraction was performed to collect the following data from the included trials: patients’ general characteristics, intervention, control group, outcomes, study, and follow-up. Two reviewers independently extracted the relevant data from the published articles. Outcomes such as pain scores, total blood loss, range of motion, functional KSSs, clinical KSSs, and operating time were abstracted and recorded on a form (Table 1). In studies in which data were incomplete or unclear, attempts were made to contact investigators for clarification. All data were extracted by two independent reviewers, and disagreements were resolved by discussion.

**Table 1:** The general characteristics of the included studies. *1* pain scores, *2* total blood loss, *3* range of motion, *4* clinical KSSs, *5* functional KSSs, *6* operating time *NS* not stated, *RCTs* randomized controlled trials

Author	Mean age	Male patients (%)	Cases	Intervention	Control	Outcomes	Follow-up	Study
Dong 2016 [11]	65	43.5	60	Synovectomy	Synovium-retaining	3,4,5,6	12 months	RCTs
Hua 2015 [12]	70	55.6	187	Synovectomy	Synovium-retaining	1,2,3,4,5,6	At discharge	RCTs
Kilicarslan 2011 [13]	68	34.7	100	Synovectomy	Synovium-retaining	1,2,3,4,5,6	6 months	RCTs
Li 2014 [14]	67	44.1	90	Synovectomy	Synovium-retaining	3,4,5,6	NS	RCTs
Ning 2014 [15]	67	52.4	374	Synovectomy	Synovium-retaining	1,2,3,4,5,6	NS	RCTs
Shaoning 2014 [16]	58	48.2	187	Synovectomy	Synovium-retaining	1,2,3,4,	NS	RCTs
Tanavalee 2011 [17]	70	41.6	67	Synovectomy	Synovium-retaining	1,2,3,4,5,6	3 months	RCTs
Yang 2016 [18]	67	45.4	118	Synovectomy	Synovium-retaining	1,2,3,4,5,6	NS	RCTs
Zhaoning 2013 [5]	65	38.7	187	Synovectomy	Synovium-retaining	1,2,3,4,5,6	12 months	RCTs
Hu 2017 [19]	70	42.5	105	Synovectomy	Synovium-retaining	1,2,3,4	3 months	RCTs

### Statistical analysis

The statistical analysis was conducted using Stata 12.0 software. Continuous outcomes (total blood loss, range of motion, functional KSSs, clinical KSSs, and operating time) were expressed as weighted mean differences (WMD) and 95% confidence interval (CI). Pain scores were expressed as standard mean difference (SMD) and 95% CI as they use different methods to assess the pain intensity. *P* < 0.05 was considered statistically significant. Heterogeneity was assessed with the *χ*^2^-based Q testing. If there was significant heterogeneity (*P* < 0.1), we selected a random-effects model to pool the data. If not, a fixed effects model was used. Publication bias was tested using funnel plots and Begg’s test (*P* > 0.05 was identified as no publication bias).

## Results

### Literature characteristics

**Fig. 1:**
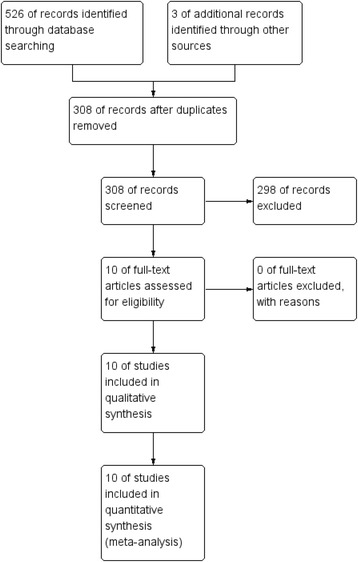
Flowchart of study search and inclusion criteria

Figure 1 presents the selection of the included studies. In the initial search, we identified 526 potentially relevant studies and 3 additional records through other sources. Among the 529 articles, 221 duplicates were removed by Endnote Software (Version X7, Thompson Reuters, CA, USA). According to the inclusion criteria, 298 studies were excluded after reading the titles and abstracts. Finally, we included 10 clinical trials in the meta-analysis [5, 11–19].

### Quality assessment

**Fig. 2:**
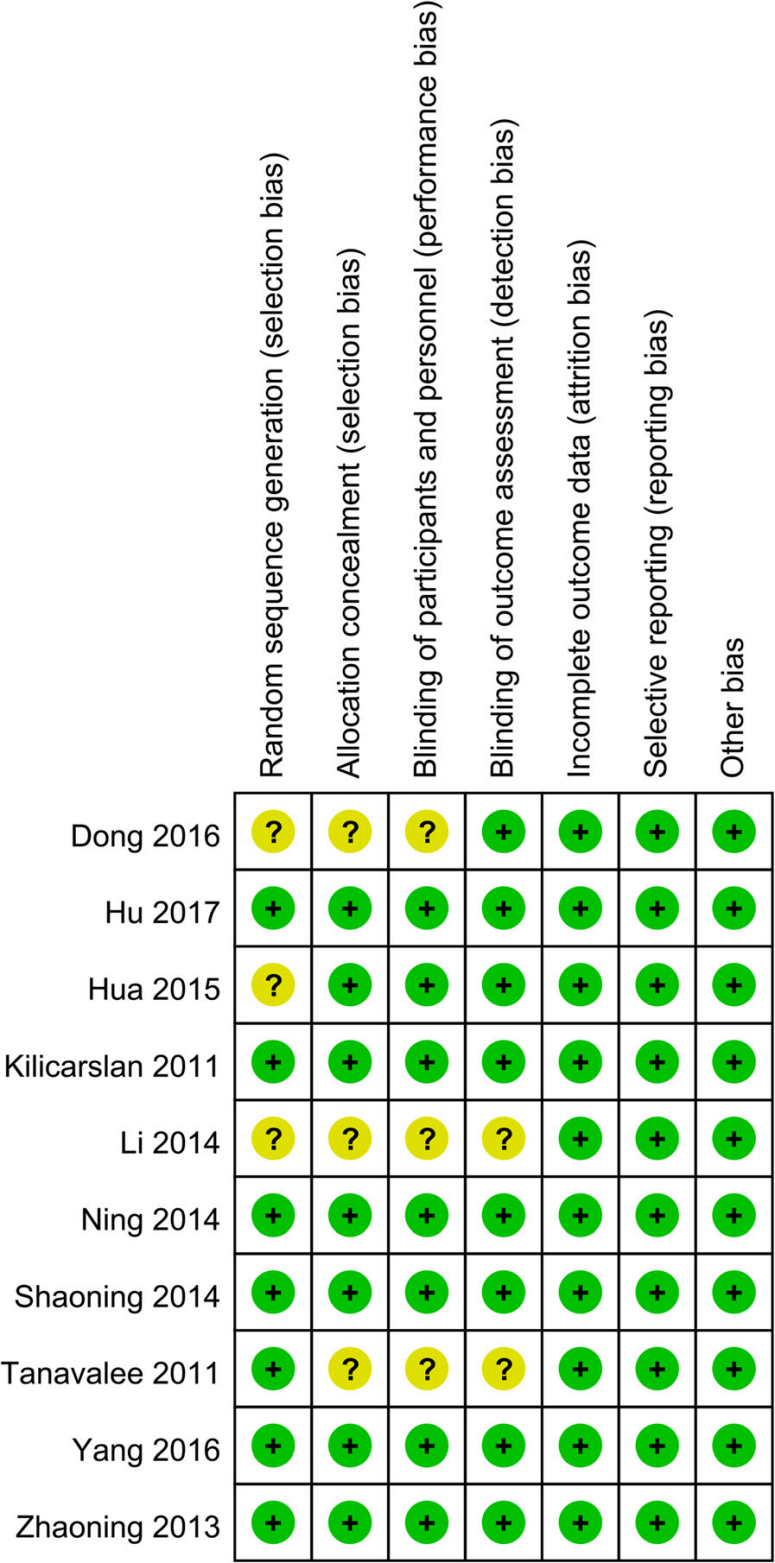
Risk-of-bias summary of included randomized controlled trials. +, no bias; −, bias; ?, bias unknown

**Fig. 3:**
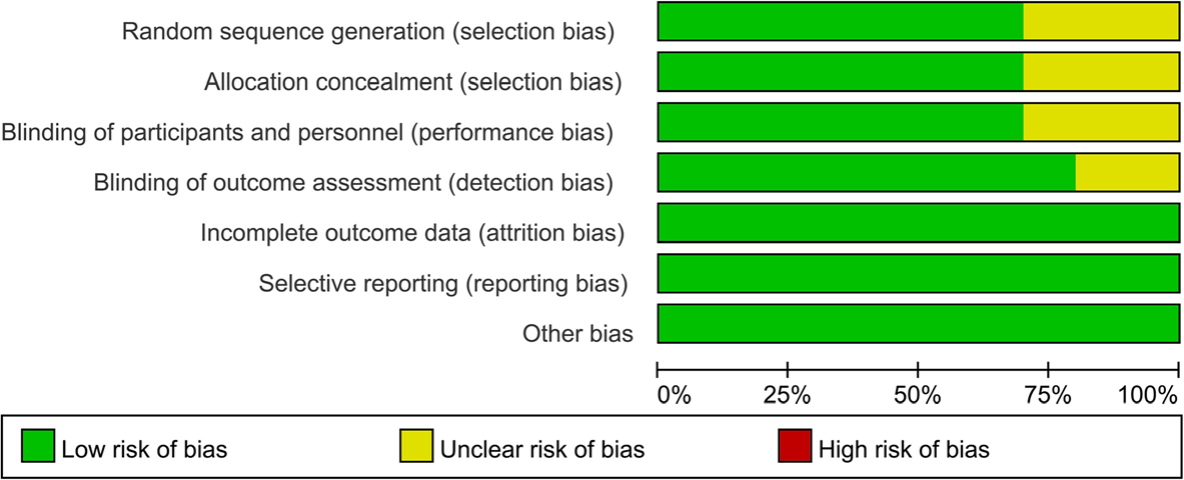
The risk of bias graph

The kappa value between the two reviewers was 0.803, which indicated that there was good agreement between the two reviewers. The quality assessment of the included studies is summarized in Figs. 2 and 3. Seven studies refer to the random sequence generation and the rest three studies did not state the random sequence generation and identify as unclear risk of bias. One of the seven studies refers to the allocation concealment and identifies as low risk of bias. Other risks of bias are all with unclear risk of bias. Two studies did not refer to the blinding of the participants and outcome assessment. Other biases are all with low risk of bias.

### Results of meta-analysis

#### Pain scores

**Fig. 4:**
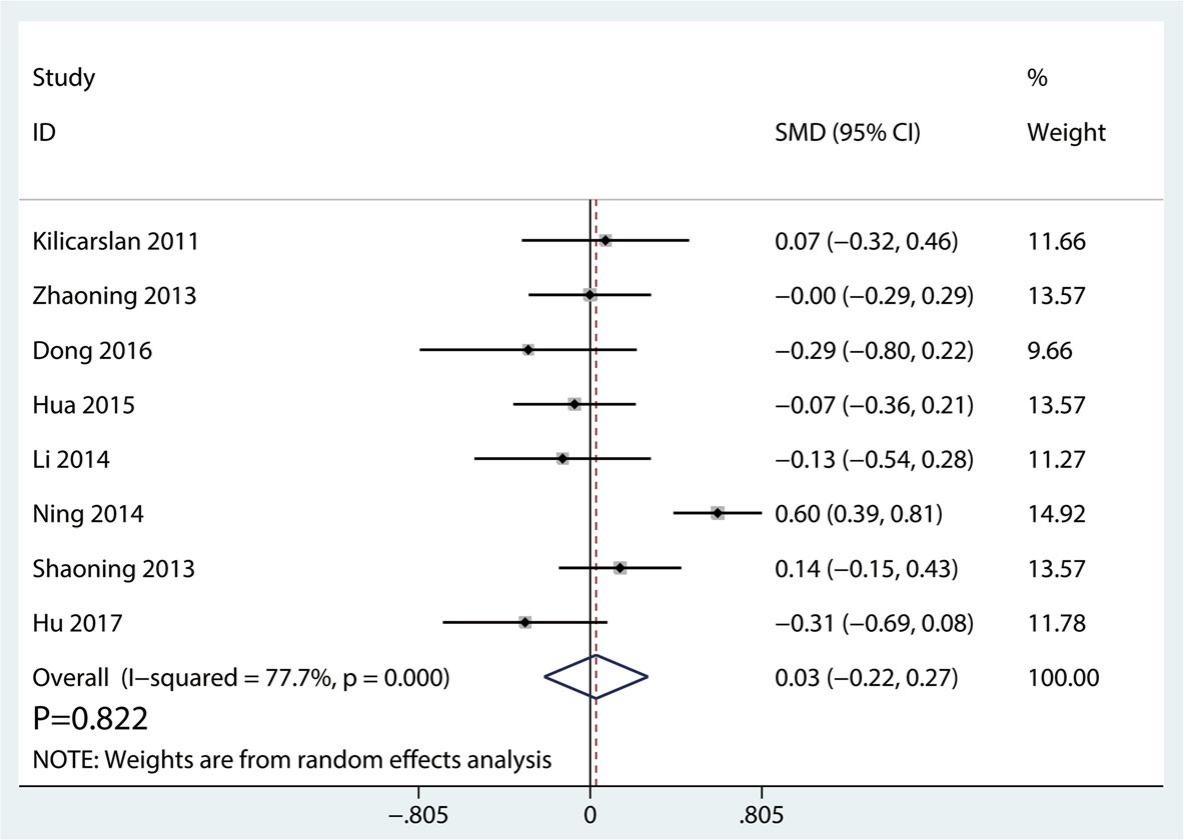
Forest plots of the included studies comparing the pain scores

We obtained usable data on pain scores from eight trials including 1290 knees. As depicted in Fig. 4, there was significant heterogeneity (*I*^2^ = 77.7%, *P* = 0.000), and thus, we used a random-effects model. And the pooled results indicated that there was no significant difference between synovectomy group and control group in terms of the postoperative pain scores (SMD = 0.03, 95% CI − 0.22, 0.27, *P* = 0.822).

#### Total blood loss

**Fig. 5:**
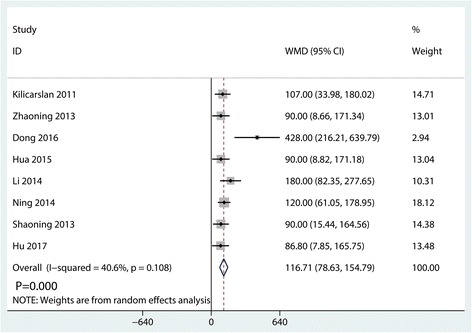
Forest plots of the included studies comparing the total blood loss

Total blood loss was reported in eight studies, and the pooled results indicated that the synovectomy group was associated with an increase of the total blood loss (WMD = 116.71, 95% CI 78.63, 154.79, *P* = 0.000, Fig. 5). A random-effects model was used because statistical heterogeneity was found between the studies (*I*^2^ = 40.6%, *P* = 0.108).

#### Range of motion

**Fig. 6:**
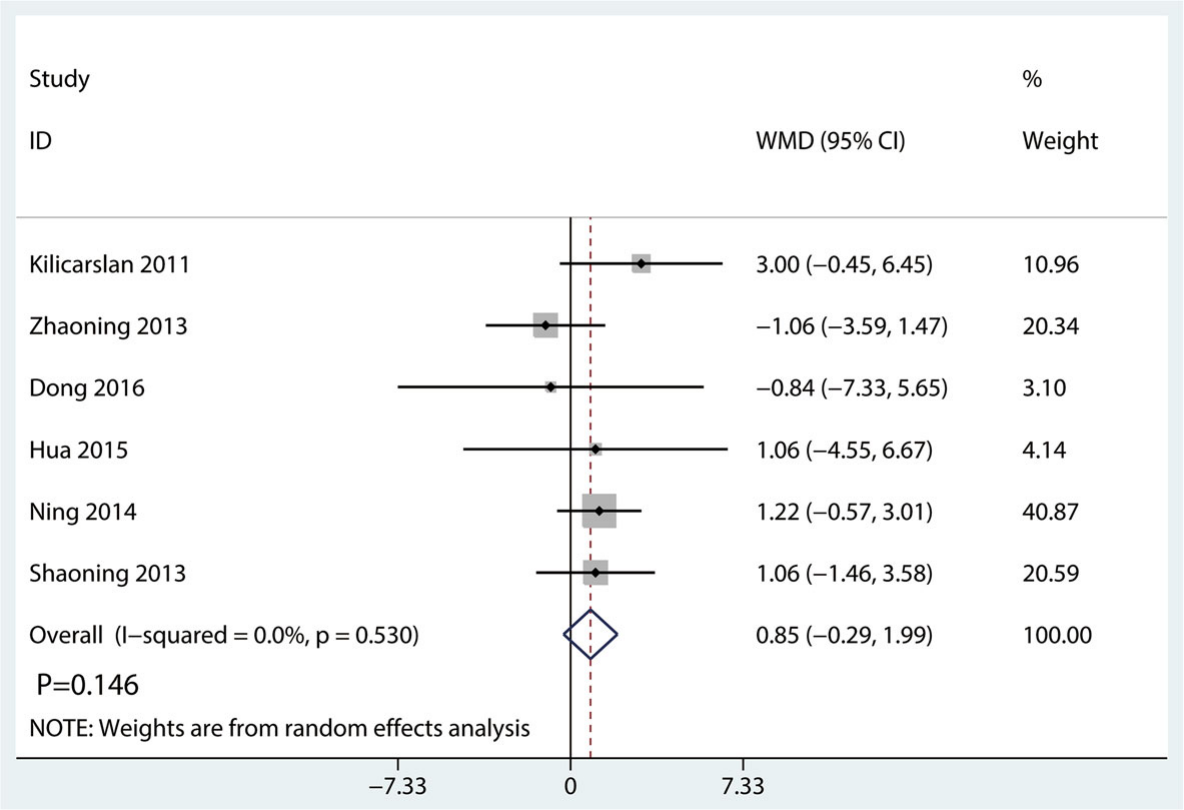
Forest plots of the included studies comparing the range of motion

Range of motion was mentioned in six studies; the pooled results showed no significant heterogeneity (*I*^2^ = 0.0%, *P* = 0.530). And the pooled results indicated that there was no significant difference between the synovectomy group and control group in terms of the range of motion (WMD = 0.85, 95% CI − 0.29, 1.99, *P* = 0.146, Fig. 6).

#### Clinical KSS

**Fig. 7:**
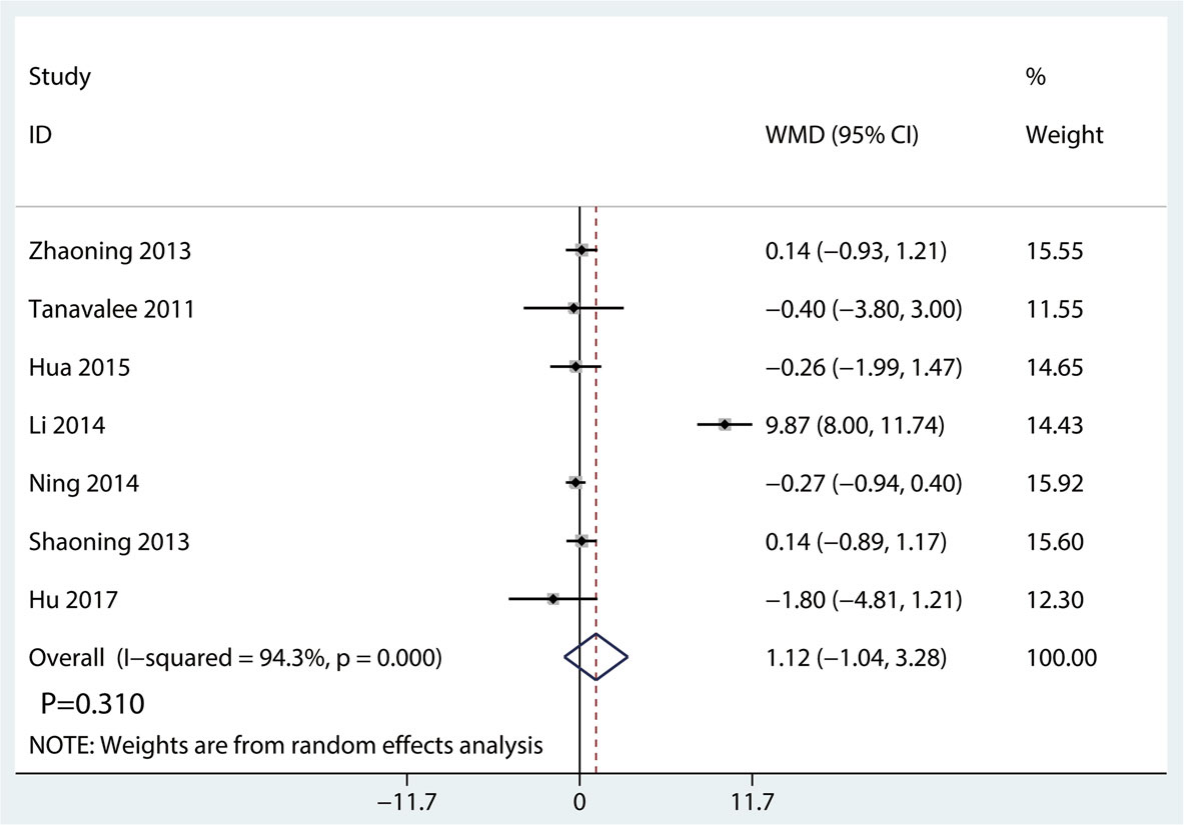
Forest plots of the included studies comparing the clinical KSS

Clinical KSSs were reported in seven studies, and the pooled results indicated that there was no significant difference between the synovectomy group and control group in terms of the clinical KSS (WMD = 1.12, 95% CI − 1.04, 3.28, *P* = 0.310, Fig. 7).

#### Functional KSS

**Fig. 8:**
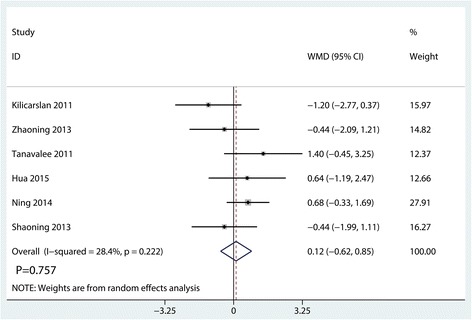
Forest plots of the included studies comparing the functional KSS

Functional KSSs were reported in six studies, and the pooled results indicated that there was no significant difference between the synovectomy group and control group in terms of the functional KSS (WMD = 0.12, 95% CI − 0.62, 0.85, *P* = 0.757, Fig. 8).

#### Operating time

**Fig. 9:**
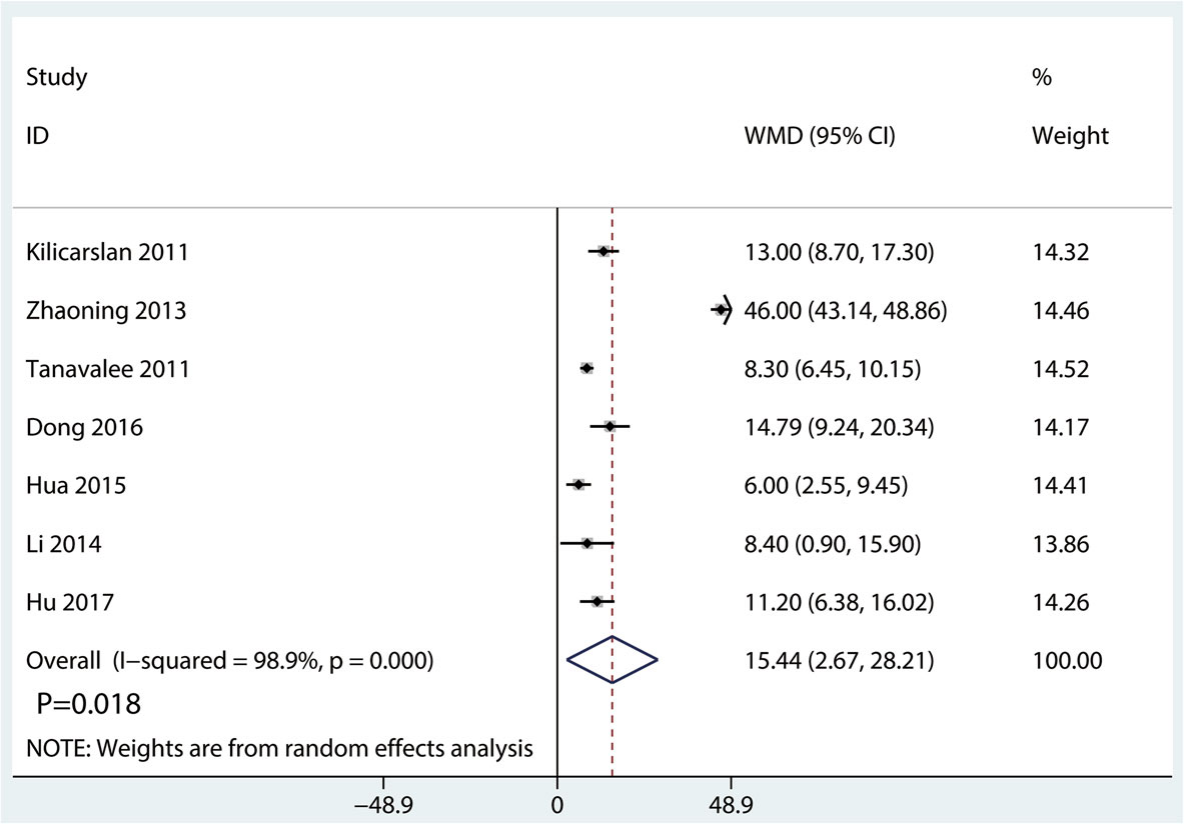
Forest plots of the included studies comparing the operating time

Operating time was reported in three studies, and the pooled results indicated that synovectomy was associated with an increase of the operating time (WMD = 15.44, 95% CI 2.67, 28.21, *P* = 0.018, Fig. 9).

#### Funnel plot and sensitivity analysis

**Fig. 10:**
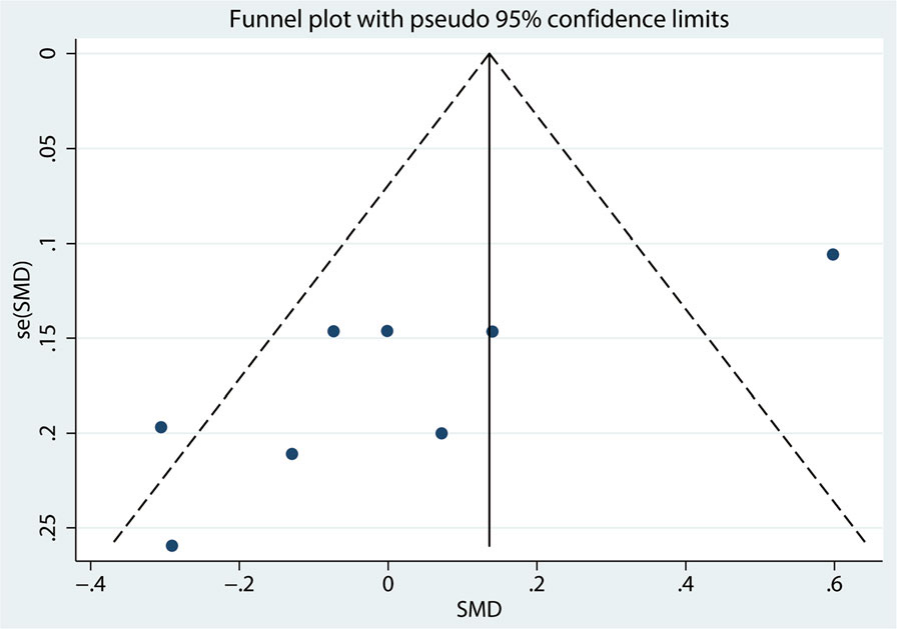
Funnel plot of the pain scores

**Fig. 11:**
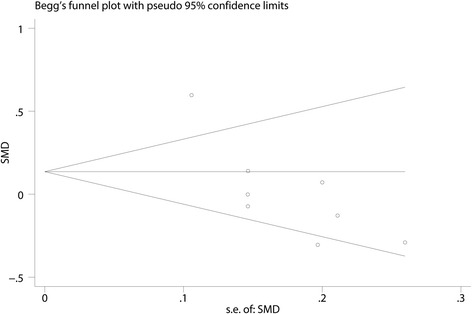
Begg’s test of the pain scores

**Fig. 12:**
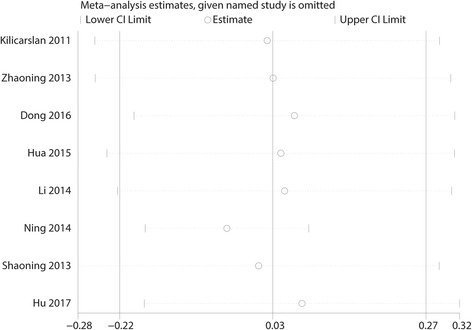
Sensitivity analysis of the pain scores

Funnel plot and Begg’s test of pain scores are seen in Figs. 10 and 11, respectively. Results show that there was a potential publication bias for the pain scores (*P* = 0.012). Sensitivity analysis shows that after excluding the studies one by one, the entire results were not changed (Fig. 12).

## Discussion

Current meta-analysis indicated that, compared with control group, synovectomy in conjunction with TKA for primary OA provides no clinical benefits. Results have shown that synovectomy will cause more blood loss and operating time. And there was no significant difference between the pain scores, range of motion, and postoperative KSS.

A major strength of the current meta-analysis was that we included 10 RCTs and increased the credibility of the meta-analysis. Kooner et al. [8] performed a meta-analysis and found that there is currently no evidence to support the use of synovectomy in TKA for primary OA, as it provides no clinical benefit and increases postoperative blood loss. However, in that meta-analysis, only three RCTs were included and the author concluded in his meta-analysis that the major limitation of this review was the lack of studies included for analysis, as well as a lack of raw data. In the current meta-analysis, we included 10 RCTs and thus provided relative confidence level.

Fernandez-Madrid et al. [20] suggest that synovitis occurs in OA or RA patients and might be a contributing cause of pain, and it has been proposed that surgical synovectomy can reduce pain and improve function after TKA. Most evidence for synovectomy stems from much of the literature addressing TKA in inflammatory arthropathies and other inflammatory conditions [21, 22]. Most studies have reported favorable results following synovectomy combined with other specific surgical procedures [23]. Inflammation in OA is unlikely the cause of persistent pain postoperatively. The potential reason was patellar maltracking, improper placement of the prosthesis, and infection.

Functional and clinical KSSs were used to assess the postoperative knee function. However, we did not find any benefit of synovectomy for patients prepared for TKA. Zhaoning et al. [5] reported that postoperative recovery was not affected by retention or excision of the synovial membrane of the knee joint. Krackow [6] recommended that as little synovium as possible should be removed, and Yasgur et al. [24] recommended that only sufficient synovium should be excised to ensure adequate visualization. In current clinical practice, postoperative rehabilitation exercise was the main factor that affected the functional and clinical KSS. Since the postoperative rehabilitation was similar, thus no significant difference was observed in these two groups.

As for total blood loss and operating time, we found that synovectomy will increase the total blood loss and the operating time. Kooner et al. [8] also found that postoperative blood loss was significantly lower in synovium-retaining TKA group and the difference was statistically significant (MD = 99.41; 95% CI, 45.08–153.75). Pooled results in this meta-analysis indicated that the synovectomy group was associated with an increase of the total blood loss (WMD = 116.71, 95% CI 78.63–154.79, *P* = 0.000).

There were several limitations in this meta-analysis: (1) only 10 RCTs were included, which might have affected the precision of the effect size estimations; (2) follow-up in the included studies ranged from 24 h to 6 month, and the relatively short-term follow-up may underestimate the complication rate; (3) perioperative nursing may be different and thus may cause the heterogeneity; (4) the follow-up duration in the included studies were relatively short and long-term follow-up was needed; and (5) publication bias existed in the pain scores and may affect the final results.

## Conclusion

In conclusion, current evidence indicates that synovectomy has no effects on the final clinical outcomes for OA patients undergoing TKA. It will increase the total blood loss and the operating time during TKA. Because the sample size and the number of included studies were limited, a multi-center RCT is needed to identify the effects of synovectomy in reducing pain after TKA.

## Abbreviations

CI: Confidence interval
KSS: Knee Society Score
OA: Osteoarthritis
PRISMA: Preferred Reporting Items for Systematic Reviews and Meta-Analyses
RA: Rheumatoid arthritis
RCTs: Randomized controlled trials
SMD: Standard mean difference
TKA: Total knee arthroplasty
WMD: Weighted mean difference
